# Supplementary material for: SpatialCorr identifies gene sets with spatially varying correlation structure
Source: Cell Rep Methods. 2022 Dec 13;2(12):100369. doi: 10.1016/j.crmeth.2022.100369 (PMC9795364; doi:10.1016/j.crmeth.2022.100369)
Supplement: Document S1. Figures S1–S4 [file mmc1.pdf]

Cell Reports Methods, Volume *2*

Supplemental information

SpatialCorr identifies gene sets with spatially varying correlation structure

Matthew N. Bernstein, Zijian Ni, Aman Prasad, Jared Brown, Chitrasen Mohanty, Ron Stewart, Michael A. Newton, and Christina Kendziorski

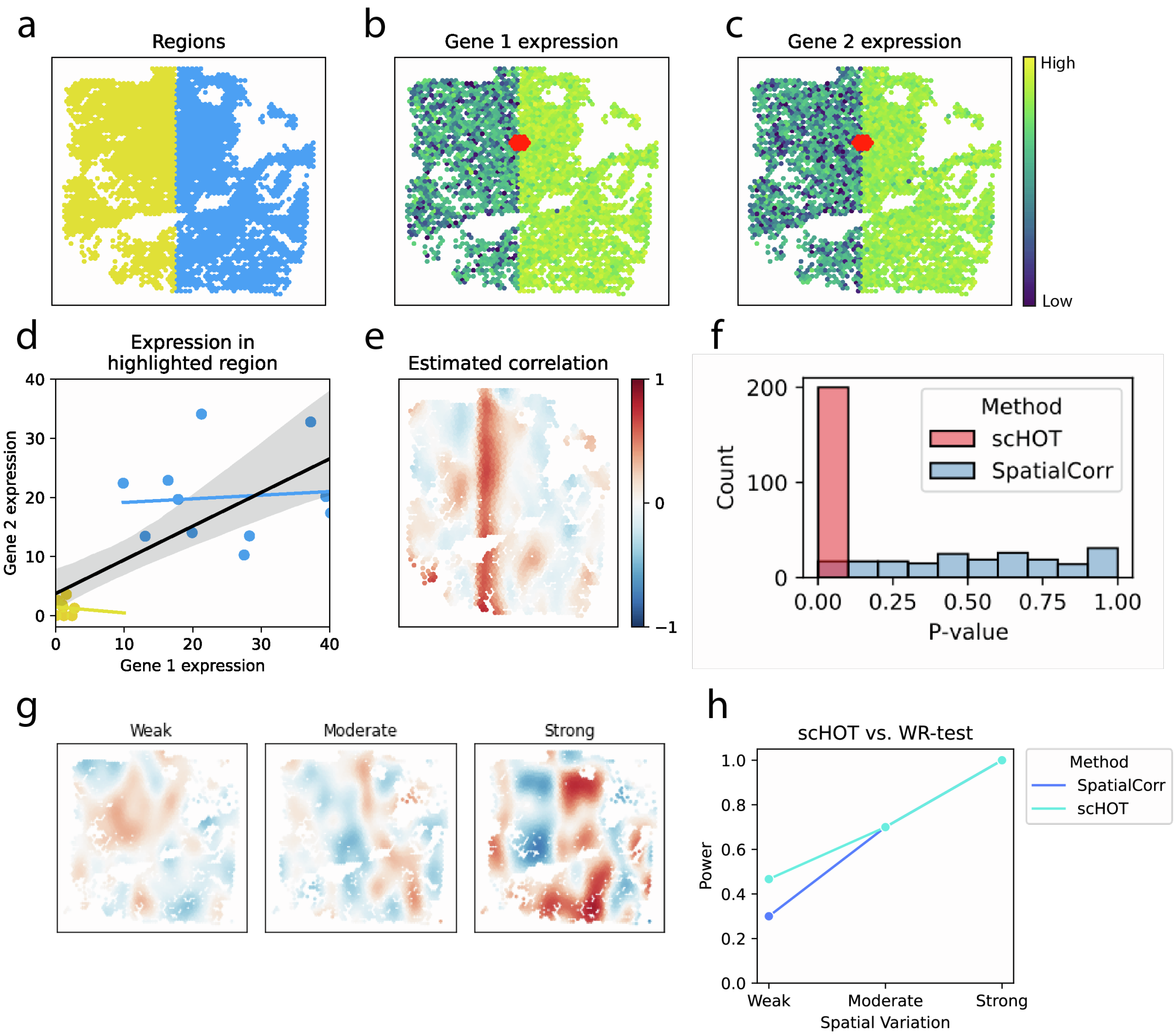

Supplementary Figure S1. Comparing SpatialCorr to scHOT. Related to Figure 2. We depict an example dataset from Sim V in which the slide is (**a**) comprised of two regions (yellow and blue) where the two genes' mean expression differs between the two regions (**b**,**c**), but the correlation between these two genes is zero within each region. We highlight a region of spots (red) along the border between the two regions and show a scatterplot of the expression values for the spots within this region (**d**). Points are colored according to the region to which they belong. The yellow line shows the least-squares regression line for points in the yellow region; the blue line shows the regression line for points in the blue region. The slopes of each are near zero which is expected since the genes are not correlated within region. Also shown in solid black is the least-squares regression line fit to points from both regions; 95% error regions are generated from bootstrapping. Here, the genes appear to be correlated due to differences in mean expression. (**e**) The kernel estimates of the correlation at each spot shows a band of high correlation along the border between the two regions. (**f**) The distribution of p-values produced by scHOT and SpatialCorr when run on 200 simulated datasets from Sim V. (**g**) Heatmaps depicting three

examples of the latent spatially varying correlation patterns generated for each of the three correlation levels used in Sim VI to compare scHOT and SpatialCorr in the setting in which there are no SV genes. (**h**) The average power of the WR-test and scHOT on Sim VI is shown for varying correlation levels (averages taken over 30 simulated datasets).

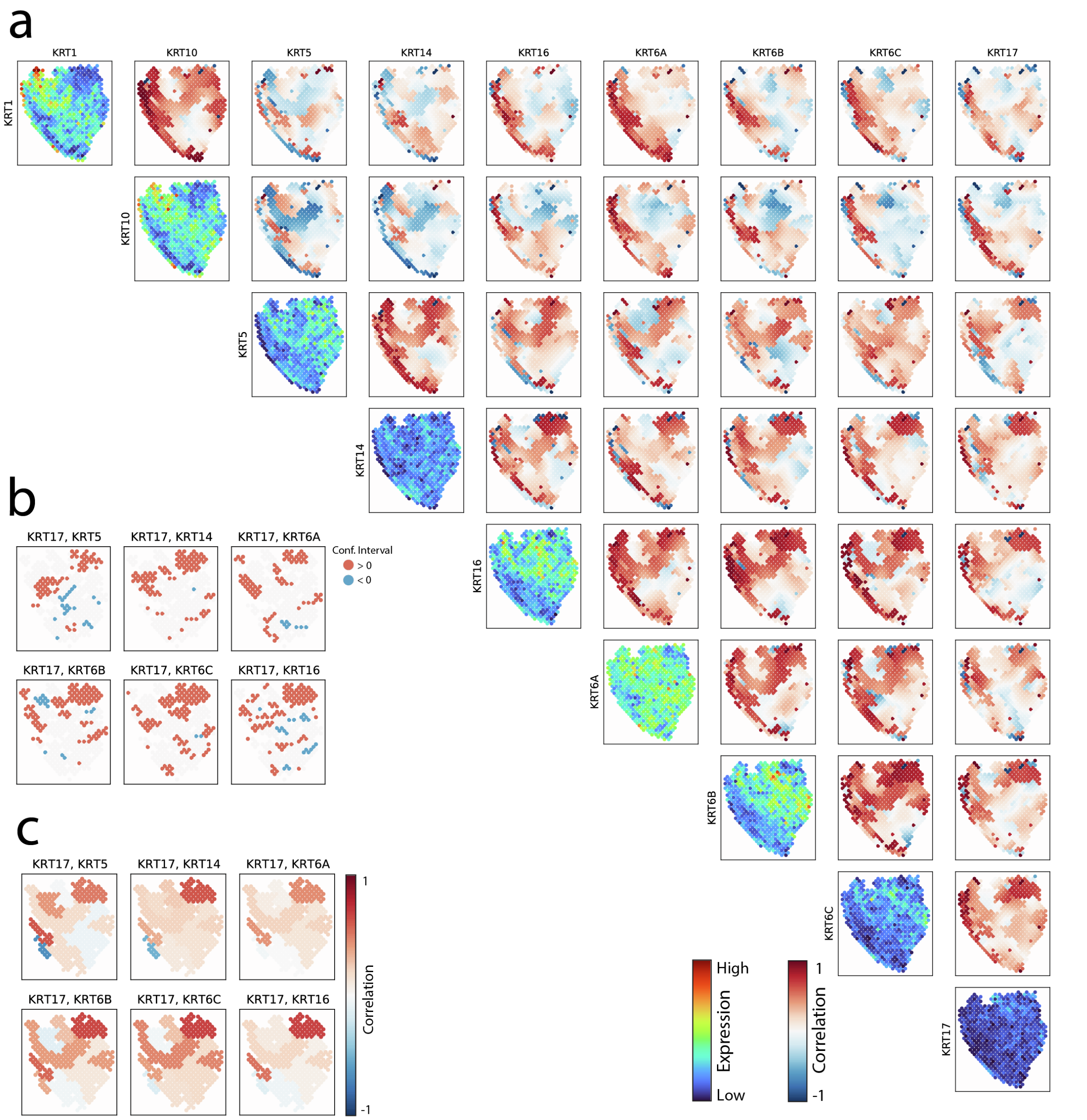

Supplemental Figure S2. Estimated correlation between keratin genes in Patient 10. Related to Figure 3. (**a**) Off-diagonal heatmaps depict the spot-specific kernel estimates of correlation between all pairs of keratin genes in the "Keratinocyte Differentiation" GO category. Heatmaps along the diagonal depict the spot-specific expression of each gene. No effective-neighbors filter was applied. (**b**) In addition to calculation and visualization of kernel estimates of correlation, the SpatialCorr package also enables one to calculate and visualize approximate confidence intervals around those estimates. A spot is colored red if the 95% confidence interval around the correlation estimate lies above zero. A spot is colored blue if the interval lies below zero. Otherwise, the spot is colored grey. (**c**) SpatialCorr also calculates and visualizes the correlations estimated on a per-region basis using all spots in a region.

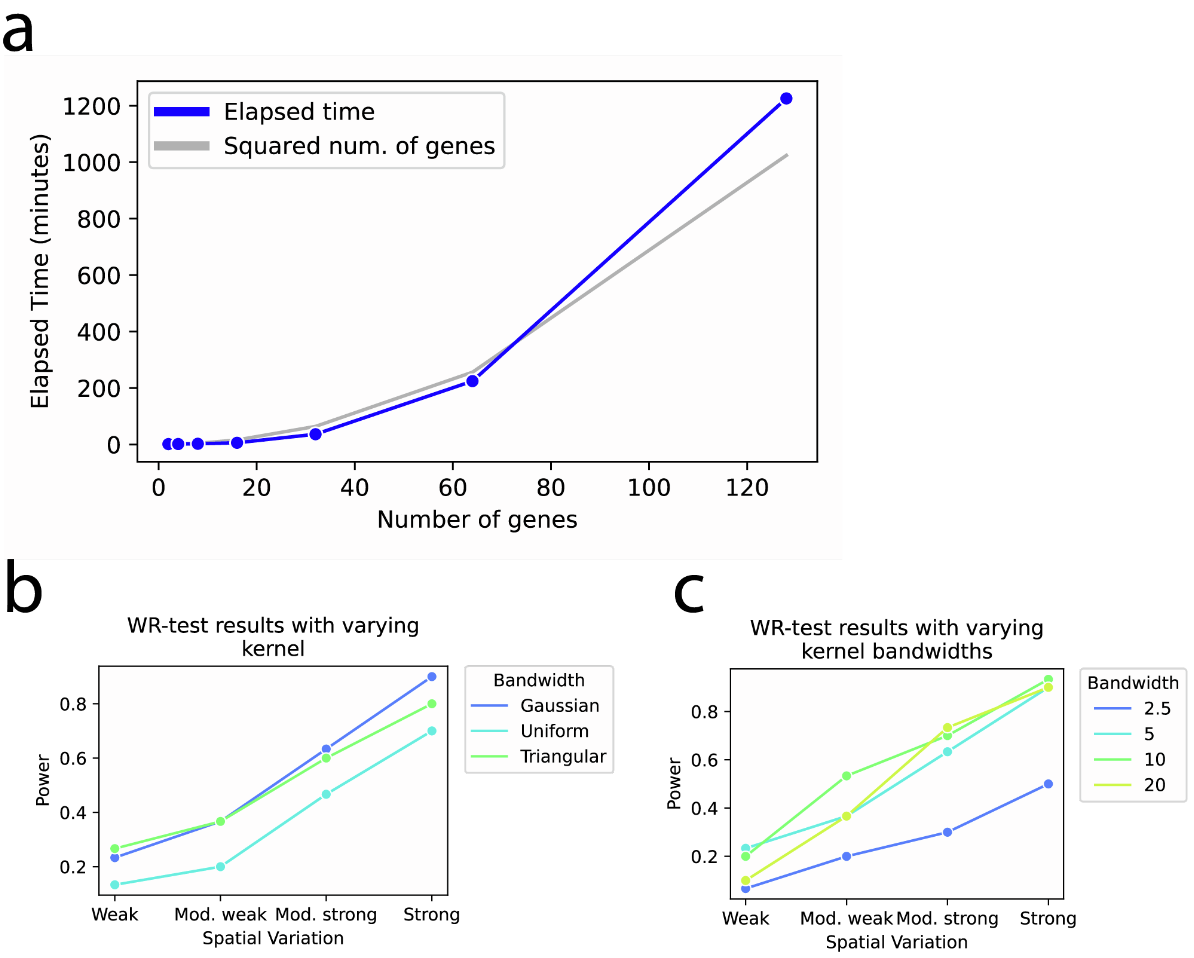

Supplemental Figure S3. Assessment of performance when varying the kernel function and number of input genes. Related to STAR Methods. (**a**) The elapsed time taken to run SpatialCorr's WR-test on the SpatialLIBD data using only five permutations per execution with varying sized input gene sets (blue line). In grey, we plot the scaled square of the number of input genes. (**b**) The average power of the WR-test is shown for varying correlation and kernel functions (average taken over 30 simulated datasets generated under Sim I). (**c**) The average power of the WR-test is shown for varying correlation and kernel bandwidth parameter values using the Gaussian kernel (average taken over 30 simulated datasets generated under Sim I).

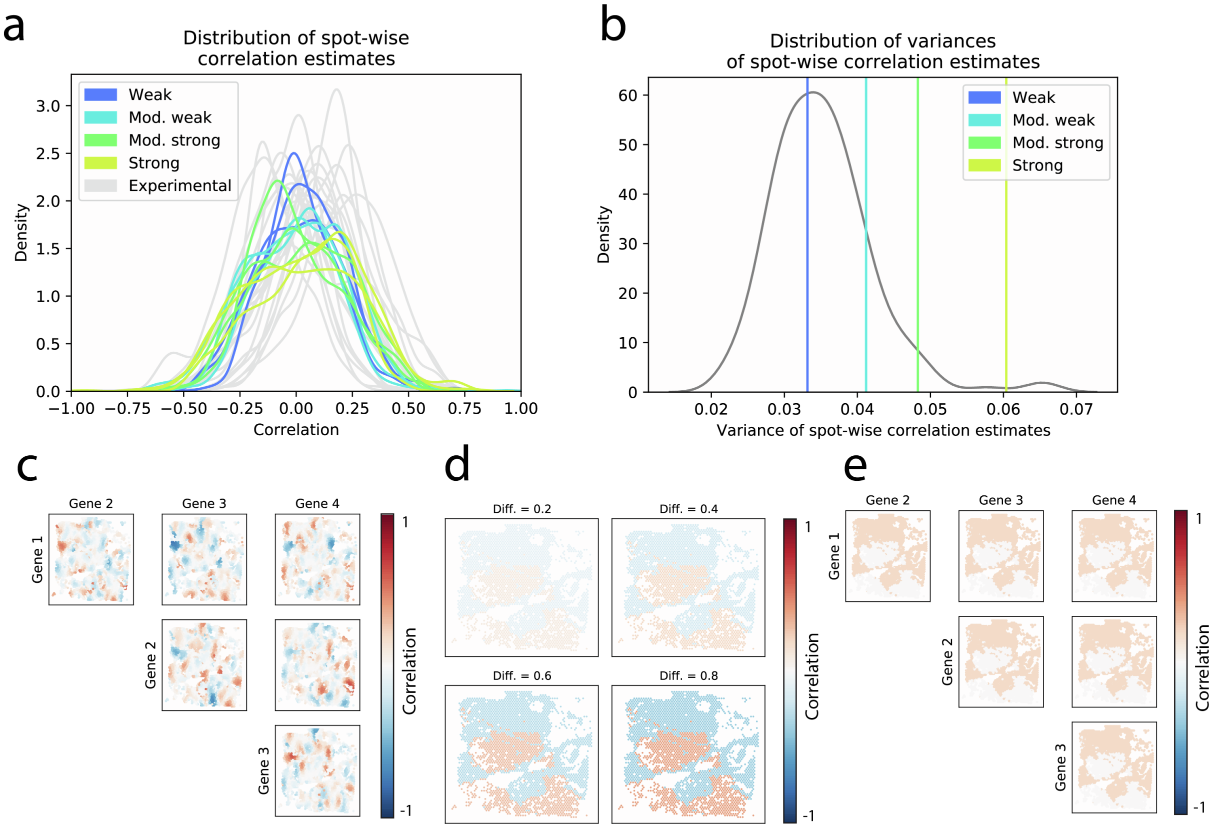

Supplemental Figure S4. Choosing simulation parameters. Related to STAR Methods. (**a**) The kernel density estimated distributions of the spot-wise correlations in simulated (colorful) and experimental (grey) datasets. Specifically, we sample 10 random pairs of genes whose expression falls within ranks 40-60 from the experimental data according to median UMI count, estimate each-spot's correlation between each pair using Gaussian kernel estimation, and for each pair, plot the distribution of those per-spot correlations across the slide. For each latent correlation level (weak, moderately weak, moderately strong, and strong), we simulated three datasets and repeat this procedure. (**b**) For each gene pair within ranks 40-60 in the experimental data according to median UMI count, we estimate each spot's correlation using Gaussian kernel estimation, and for each pair, compute the variance of those spot-wise correlation estimates across the slide. Shown is the distribution of these empirical variances across all gene pairs (grey). For each simulated latent correlation level (weak, moderately weak, moderately strong, and strong), we repeat this procedure, calculate the average variance in simulated datasets (taken over 10 replicates), and plot these average values above (colorful vertical lines). (**c**) Heatmaps showing examples of the spot-wise latent correlations used to simulate four genes in Sim II, (**d**) pairs of genes in Sim III, and (**e**) four genes in Sim IV.
